# Einlagenversorgung in der Therapie des Knicksenkfußes: Prospektive, randomisierte, doppelt verblindete, placebokontrollierte Studie zum Vergleich von sensomotorischen und stützenden Einlagen

**DOI:** 10.1007/s00132-024-04589-1

**Published:** 2024-12-27

**Authors:** Alexander Pascal-Laurent Schmitt, Kira-Henriette Liebau, Alexander Hamm, Wolfram Mittelmeier, Christoph Schulze

**Affiliations:** 1 Orthopädische Klinik und Poliklinik, Universitätsmedizin Rostock, Doberaner Str. 142, 18057 Rostock, Deutschland; 2 Zentrum für Sportmedizin der Bundeswehr, Dr.-Rau-Allee 32, 48231 Warendorf, Deutschland; 3 Bundeswehr Facharztzentrum Hohe Düne, Hohe Düne 30, 18119 Rostock, Deutschland; 4 Universitätsinstitut für Physikalische Medizin und Rehabilitation, Paracelsus Medizinische Universität, Müllner Hauptstr. 48, 5020 Salzburg, Österreich

**Keywords:** Erwachsene, Fußdeformität, Fußmuskeltraining, Auflagefläche, Funktionalität, Adults, Foot deformity, Foot muscle exercises, Foot contact area, Foot function

## Abstract

**Hintergrund:**

Bei Erwachsenen erfolgt die Therapie des flexiblen symptomatischen Knicksenkfußes konservativ mit stützenden Einlagen, während sensomotorische Einlagen bei unzureichender Datenlage umstritten sind.

**Fragestellung:**

Untersuchung der Wirksamkeit sensomotorischer und stützender Einlagen bei Erwachsenen.

**Material und Methoden:**

Bei 73 Patienten wurden zusätzlich zu Fußgymnastik im Rahmen einer doppelt verblindeten, prospektiven, randomisierten placebokontrollierten Studie stützende, sensomotorische oder Placeboeinlagen unter Nutzung von Numerische Rating-Scala, Foot and Ankle Disability Index sowie Pedobarographie und Valgus-Index über 3 Messpunkte in einem Jahr miteinander verglichen. Die statistische Auswertung erfolgte mittels ANOVA mit Messwiederholungen.

**Ergebnisse:**

Der Valgus-Index wurde bei stützenden Einlagen signifikant größer. Die Auflagefläche des Fußes konnte in der Verlaufskontrolle nur bei sensomotorischen Schuheinlagen in statischer und dynamischer Messung signifikant reduziert werden. Stützende Einlagen führten zu einer schnelleren Schmerzreduktion, jedoch ohne Reduktion der Auflagefläche. In der Funktionalität zeigten sich keine relevanten Unterschiede.

**Schlussfolgerungen:**

Die Reduktion der Auflagefläche in Verbindung mit sensomotorischen Einlagen zeigt das Potenzial zur muskulären Adressierung des flexiblen Knicksenkfußes. Nachteile gegenüber anderen Versorgungen zeigten sich nicht. Stützende Einlagen führten schneller zur Reduktion von subjektiven Beschwerden, schwächen aber scheinbar die Fußwölbung-stützende Muskulatur. Längerfristig erscheint die konsequente Durchführung von Fußmuskeltraining ebenfalls wirksam zu sein, da auch das Tragen von Placeboeinlagen ohne signifikante biomechanische Änderungen zu einer Verbesserung des subjektiven Wohlbefindens führte.

## Abstract (en)

**Background:**

In adults, flexible symptomatic flat foot is treated conservatively with supportive foot orthoses. Sensorimotor foot orthoses, however, are controversial due to insufficient data.

**Purpose:**

Comparison of the effectiveness of sensorimotor and supportive foot orthoses in adults.

**Material and methods:**

In 73 patients, in addition to foot gymnastics, supportive, sensorimotor or placebo insoles were compared over 3 measurement points during 1 year as part of a double-blind, prospective, randomised placebo-controlled clinical trial using Numeric Rating-Scala, the Foot and Ankle Disability Index, as well as pedobarography and valgus index. The statistical analysis was performed using ANOVA with repeated measures.

**Results:**

The valgus index increased significantly with supportive foot orthoses. In the follow-up, the foot contact area was only significantly reduced with sensorimotor foot orthoses in static and dynamic measurements. Supportive foot orthoses led to a faster reduction in pain, but without reducing the contact area of the foot. There were no relevant differences in functionality.

**Conclusions:**

The reduction of the contact surface in combination with sensorimotor foot orthoses shows the potential for muscular addressing of the flexible flat foot. There were no disadvantages compared to other treatments. Supportive foot orthoses led to a faster reduction in subjective complaints, but appear to weaken the muscles supporting the arch of the foot. In the longer term, consistent foot muscle training also appears to be effective, as wearing placebo foot orthoses also led to an improvement in subjective well-being without significant biomechanical changes.

## Hinführung

Der flexible Knicksenkfuß wird konservativ häufig mit stützenden Einlagen versorgt. Sensomotorische Einlagen werden hier neuerdings ebenfalls therapeutisch eingesetzt. Die Datenlage zur Wirksamkeit beider Therapieformen ist eingeschränkt. Insbesondere vergleichende Arbeiten sind aufgrund der Heterogenität der verwendeten Produkte wenig verfügbar. Ebenso wenig werden biomechanische Effekte bisher konsequent und einheitlich berichtet. Diese Arbeit soll durch die Kombination von klinischen Scores und biomechanischen Messungen zu einer besseren Beurteilbarkeit der Therapieangebote beitragen.

## Einleitung

Als Ursache des Knicksenkfuß bei Erwachsenen können verschiedene Punkte angeführt werden. Unter Anderem trägt eine Dysfunktion des Musculus tibialis posterior oder dessen Sehne zur Entstehung bei [1–3]. Auch eine chronische Überlastung oder eine akute Verletzung kann zu einer Schwächung des Komplexes führen und ein Absinken der Fußlängswölbung verursachen [1]. Beschwerden können hierbei im gesamten Bereich der unteren Extremität bestehen [4]. Carr et al. berichten, dass der symptomatische Knicksenkfuß zu verschiedenen Problemen wie aktivitätsbedingten Schmerzen, Ermüdung der Fußmuskulatur, Schwielen am Mittelfuß und schnellem Schuhverschleiß führen kann [4]. Soldaten mit Senkfuß haben ein höheres Risiko für das Auftreten von funktionellen Knie- und Rückenschmerzen [5]. Es wird beschrieben, dass ein beim Knicksenkfuß auftretender Rückfußvalgus zu einer Verschiebung der Lastachse führt, wobei eine Fehlbelastung des oberen Sprunggelenkes resultiert und das Risiko einer Fehlbelastung im Knie mit daraus resultierender Begünstigung der Entstehung einer Kniegelenkarthrose besteht [6, 7].

Hierbei hat die Ausprägung des Knicksenkfuß eine hohe Bedeutung [8]. Diese kann durch den Valgus-Index beschrieben werden [9]. Je höher der Valgus-Index ist, desto stärker ist der Knicksenkfuß ausgeprägt [9].

Zur konservativen Therapie eines symptomatischen Knicksenkfuß wurden Übungen für die Fußmuskulatur empfohlen und andererseits als passive Maßnahme auch Einlagen verordnet, die den Fuß stützen oder im Kindesalter auch korrigieren sollen [4]. Dabei haben die Einlagen in der Regel eine tiefe Fersenschale und stützen das Sustentaculum tali an der Längswölbung des Fußes [3]. Das Wirkprinzip zielt auf eine Reduktion von Beschwerden und ist eher symptomatisch. Eine Korrektur kann dadurch, insbesondere bei Erwachsenen nicht erreicht werden [3, 10, 11].

Sensomotorische Einlagen hingegen versuchen, aktiv auf die Muskeln im Unterschenkel, insbesondere den Musculus tibialis posterior und den Musculus peroneus longus einzuwirken [12].

Bei einer Untersuchung an 2153 Kindern wurde das Tragen von sensomotorischen und stützenden Schuheinlagen bei Knicksenkfuß bewertet. Bei 59 % der Patienten konnte nach einem halben Jahr eine Normalisierung des Gangbildes mit besserer Dynamik und Abrollbewegung beobachtet werden [13]. Die Autoren beschrieben, dass sensomotorische Schuheinlagen von den Kindern besser angenommen wurden als stützende Schuheinlagen [13]. Außerdem wurde berichtet, dass keine weitere Reduktion der Muskelstärke wie bei stützenden Schuheinlagen auftritt [13]. Bei Kindern mit Zerebralparese, Zehenspitzengängern und Kindern mit sonstigen Fußfehlstellungen konnte eine dauerhafte Korrektur des Gangbildes bei insgesamt 79 % der Patienten beobachtet werden bei guter Toleranz der Einlagen [14]. Eine Stimulation durch mechanische Vibration der an der Fußsohle befindlichen Mechanorezeptoren hat eine Beeinflussung der Balance und der Körperhaltung zur Folge und induziert Ausgleichsbewegungen des Körpers [15]. Bei Sportlern konnte gezeigt werden, dass sensomotorische Einlagen eine Änderung des motorischen Aktivierungsmusters induzieren [16]. Allerdings wurde insbesondere ein Zusammenhang von Beschwerdesymptomatik und biomechanischen Parametern im Zusammenhang mit dieser Fußdeformität unzureichend beschrieben. Vergleichende Studien zur Wirksamkeit verschiedener Einlagetypen sind wenig verfügbar. Diese Arbeit sollte vergleichend prüfen, inwieweit sensomotorische oder stützende Einlagen zu einer Reduktion der Beschwerden beitragen und ob damit einhergehend ein Einfluss auf die Biomechanik im Bereich des Fußes möglich ist.

## Material und Methoden

### Patienten

Es wurden 73 erwachsene Patienten (Soldaten, 12 weiblich, 61 männlich) mit Knicksenkfuß in die prospektive, randomisierte, placebokontrollierte Studie zwischen Juli 2016 und März 2019 aufgenommen. Die Diagnose wurde durch immer den gleichen Orthopäden klinisch gestellt. Eine radiologische Bildgebung wurde nicht durchgeführt. 15 Patienten blieben den Verlaufsbeobachtungen nach 6 oder 12 Monaten ohne Angabe von Gründen fern und wurden für die Auswertung ausgeschlossen. Tab. 1 charakterisiert die Studienpopulation. Somit konnten von 58 Patienten mit 116 Füßen (stützende Schuheinlagen [*N* = 20], sensomotorische Schuheinlagen [*N* = 23] und Placeboschuheinlagen [*N* = 15]) nachbeobachtet werden.

**Tab. 1:** Charakterisierung der Vergleichsgruppen (BMI - Body-Mass-Index)

	Sensomotorische Einlage *N* = 23	Stützende Einlage *N* = 20	Placeboeinlage *N* = 15
Alter	29,3 (±7,0)	30,6 (±5,4)	32,4 (±8,9)
BMI	27,2 (±4,6)	26,7 (±3,8)	25,5 (±3,8)
Größe in cm	1,79 (±0,08)	1,78 (±0,08)	1,80 (±0,1)
Gewicht in kg	87,52 (±19,14)	85,15 (±15,45)	82,1 (±13,5)
Geschlecht	3 Frauen/20 Männer	8 Frauen/12 Männer	1 Frau/14 Männer

Die Zuteilung der Patienten zu Therapiegruppen erfolgte anhand zuvor per Computer generierter Zufallsreihenfolge. Weder der Beobachter (immer dieselbe Person) noch die Patienten wurden darüber informiert, welcher Gruppe der Patient zugewiesen wurde (Verblindung). Einschlusskriterien waren flexibler Knicksenkfuß, Beschwerden im Bereich von Fuß und Unterschenkel im Sinne von funktionellen Schmerzen im Bereich der unteren Extremität, Ermüdung der Fußmuskulatur, Alter über 18 Jahre, keine vorhergehende Einlagenversorgung und Bereitschaft an Nachbeobachtungen teilzunehmen.

Patienten mit Erkrankungen des rheumatischen Formenkreises, Neuropathie, akutem Trauma, Arthrose der unteren Extremitäten, fixiertem Knicksenkfuß, Hohlfuß, Hallux valgus mit einem Hallux-valgus-Winkel von > 30° oder mit einer Deformität der anderen Zehen wurden ausgeschlossen. Zu jedem Messzeitpunkt nach 6 und 12 Monaten wurde die tägliche Tragedauer sowie die Compliance der Durchführung des unten beschriebenen Fußmuskeltrainings abgefragt.

Die Studie wurde in Übereinstimmung mit der Deklaration von Helsinki durchgeführt und erhielt von der örtlichen Ethikkommission ein positives Votum (Referenznummer: A2016-0009). Alle Teilnehmer gaben ihr schriftliches Einverständnis zur Teilnahme an dieser Studie, einschließlich der Erlaubnis, die Ergebnisse zu veröffentlichen.

### Schuheinlagen und Übungen

**Abb. 1:**
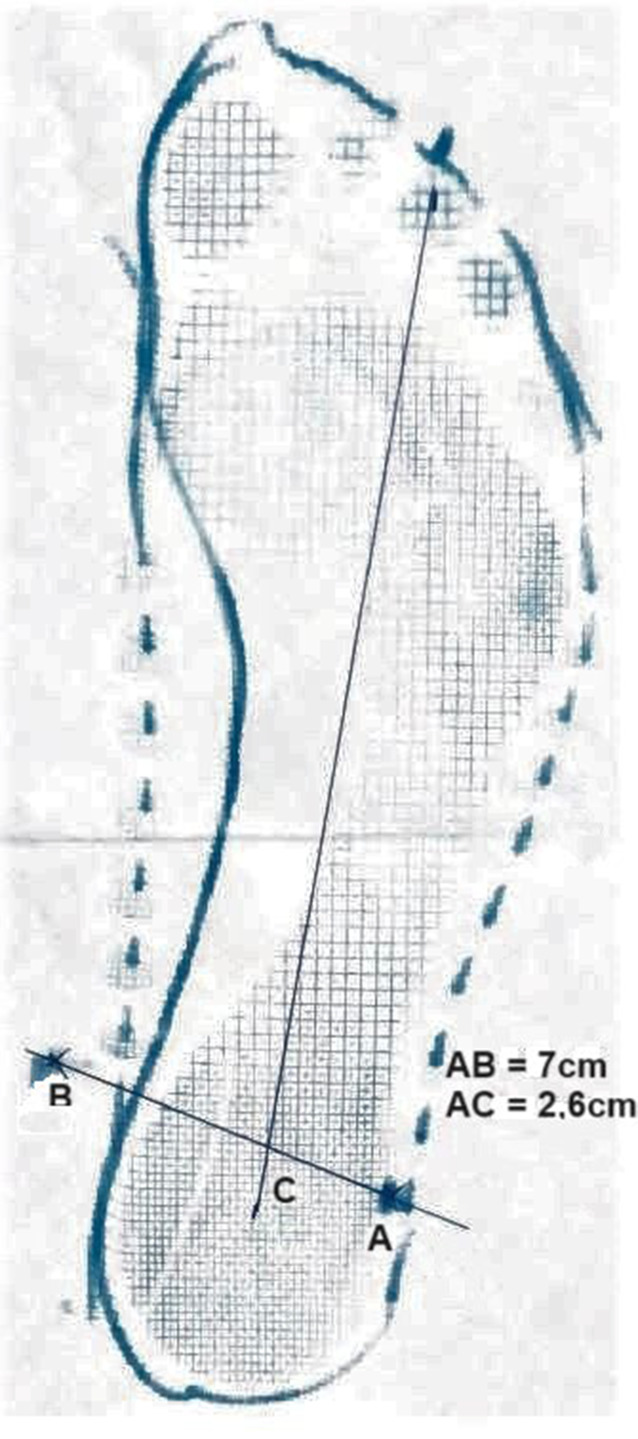
Der Blauabdruck mit Linien und Punkte zur Berechnung des Valgus-Index. (*A* Malleolus lateralis, *B* Malleolus medialis, *C* Schnittpunkt zwischen der mittleren hinteren Ferse über den Digitus pedis III). In der Formel eingesetzt: ½ × (7‑2,6) × (100/7) = 31,5; (hoher Valgus-Index). Das starke Einknicken zeigt sich bei den stark nach medial gerichteten Malleolenprojektionen

**Abb. 2:**
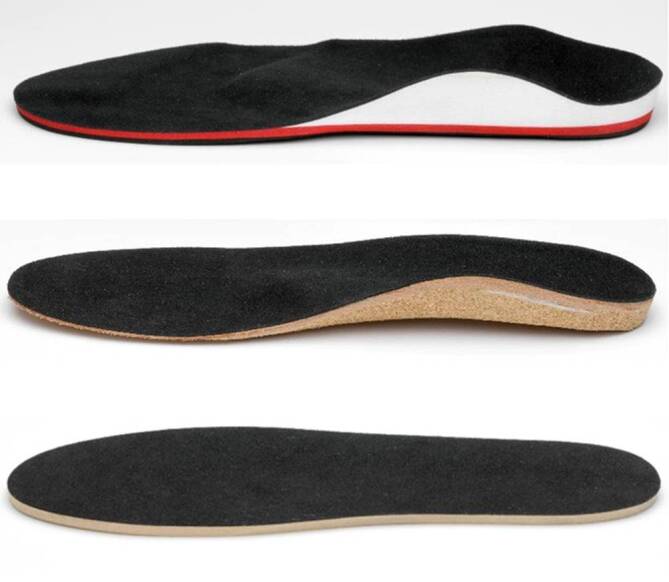
Verwendete Schuheinlagen (*oben:* sensomotorische Schuheinlage [Springer aktiv AG, Berlin, Germany]; *Mitte:* stützende Schuheinlage [Kork/Leder]; *unten:* Placeboschuheinlagen [ca. 4 mm fester, in Form geschnittener Schaumstoff])

Schuheinlagen wurden individuell auf Grundlage eines Blauabdruckes (Abb. 1) durch immer denselben Orthopädieschuhmachermeister hergestellt. Die einzelnen Exemplare (sensomotorische Einlagen [Springer aktiv AG, Berlin, Deutschland], stützende Schuheinlage [Kork, Leder] und Placeboeinlagen [ca. 4 mm fester, in Form geschnittener Schaumstoff]) werden in Abb. 2 dargestellt.

Jede Schuheinlage erhielt einen Stoffüberzug, sodass eine oberflächliche optische Ähnlichkeit erzeugt wurde, die eine bestmögliche Verblindung für Patienten und Untersucher ermöglichen sollte. Nur der Orthopädieschuhmachermeister hatte die Information der Gruppenzugehörigkeit im Rahmen der Randomisierung mit einem verschlossenen Umschlag erhalten, um die entsprechende Einlegesohle herzustellen. Die Anpassung der Schuheinlagen wurde durch immer den gleichen Orthopädieschuhmachermeister kontrolliert. Alle Probanden wurden instruiert ein festgelegtes Fußübungsprogramm für die Dauer der Nachbeobachtung von 12 Monaten durchzuführen. Die Übungen basierten auf den Prinzipien der Kurzfußgymnastik [17]. Die Patienten trugen die Schuheinlagen vornehmlich in ihren einheitlichen Dienstschuhen. Dies entspricht in der Regel eine Wochenstundenzahl von circa 40 h.

### Scores

Um Schmerzen am Fuß und an der unteren Extremität zu quantifizieren, wurde die Schmerzintensität mit der Numerischen Rating-Skala (NRS) erhoben. Um die Funktionseinschränkung des Fußes und der unteren Extremität abschätzen zu können, wurde der Foot and Ankle Disability Index (FADI) erhoben [18, 19]. Ebenso wurde die Lebensqualität nach Vorlage der EuroQol-Gruppe (EQ-5D) erhoben. Der Valgus-Index (VI), angelehnt an Rose et al., wurde auf Basis des Fußblauabdruckes ermittelt ([9]; Abb. 1). zeigt den Blauabdruck und die zur Berechnung notwendigen Maße und deren Bestimmung. Alle Parameter wurden zum Zeitpunkt der Rekrutierung (ZP0 - Ausgangsmessung), nach 6 Monaten (ZP1 - 1. Nachuntersuchung) sowie nach 12 Monaten (ZP2 - Ausgangsmessung) gemessen.

### Biomechanische Messungen

Die Auflagefläche des Fußes wurde barfuß mittels einer pedobarographischen Untersuchung (ZEBRIS Plattform Typ FDM‑S^TM^, Fa. Zebris, Isny, Deutschland) bestimmt. Die Sensordichte lag bei 1,4 Sensoren pro cm^2^. Barfußmessungen wurden binnen kürzester Zeit nach dem Ausziehen der Schuhe durchgeführt. Zusätzlich zur Messung im Stand (statisch) wurden auch Barfußmessungen in Bewegung (dynamisch) zu jedem Messzeitpunkt ausgewertet, um die maximale Auflagefläche (mittlere Standphase) zu bestimmen. Die Änderung der Auflagefläche kann als Indikator für die Aufrichtung der Fußwölbung angesehen werden. Die Messfrequenzen lagen bei 120 Hz (dynamisch) und bei 50 Hz (statisch). Für die dynamische Messung sollten alle Patienten mit einer Frequenz von 85 Schritten pro Minute (Vorgabe durch Metronom) gehen. Insgesamt wurden jeweils 7 Schritte aufgezeichnet, gemittelt und ausgewertet. Als mittlere Standphase wurde das Maximum der Kontaktfläche (in cm^2^) des Fußes auf der Pedobarographiematte angesehen. Bei der statischen Messung sollten die Patienten 10 s ohne Bewegung mit herabhängenden Armen und geradem Stand auf der Pedobarographiematte stehen. Die Auflagefläche wurde über die Zeit gemittelt und ausgewertet. Die Daten wurden softwarebasiert (Noraxon myoPressure^TM^, Fa. Noraxon, Scottsdale, AZ, USA) analysiert.

### Statistische Analyse

Die deskriptive Darstellung der Daten erfolgte für kontinuierliche Variablen auf der Grundlage der Mittelwerte mit ±1 Standardabweichung (SD). Die Normalverteilung wurde mit dem Shapiro-Wilk-Test geprüft und grafisch durch QQ-Plot nachgewiesen. Die Auswirkungen auf die klinischen Parameter und die Auflagefläche im Laufe der Zeit wurden mittels zweifaktorieller ANOVA mit Messwiederholung und Post-hoc-Bonferroni-Korrektur (SPSS V.27.0; Fa. IBM, NY, USA) durchgeführt. Das Signifikanzniveau wurde auf *p* < 0,05 festgelegt. Die Software SPSS Version 27.0. (SPSS Inc. Chicago, IL, USA) wurde für die Datenauswertung verwendet.

## Ergebnisse

### FADI

**Abb. 3:**
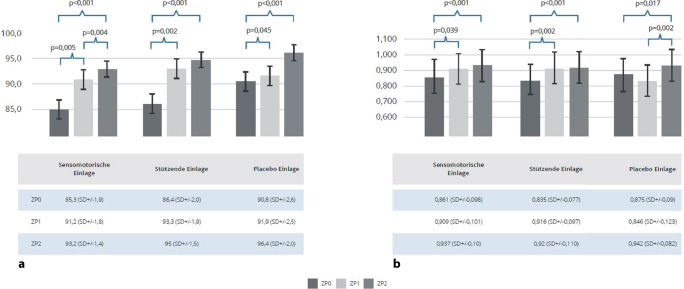
**a** Foot and Ankle Disability Index (FADI; dimensionslos); **b** EuroQol-5D (EQ5D; dimensionslos): Erhobene Werte für alle Studienarme (sensomotorische Einlage, stützende Einlage und Placeboeinlage); *ZP0 *Ausgangsmessung, *ZP1 *1. Follow-up, *ZP2 *2. Follow-up, Mittelwert mit Standardabweichung (*SD*)

Die Abb. 3a zeigt die Ergebnisse hinsichtlich der Funktionalität. Von der Ausgangsmessung zur ersten und zweiten Nachuntersuchung konnte eine signifikante Verbesserung der Symptome bei den sensomotorischen Einlagen nachgewiesen werden. Auch bei Verwendung der stützenden Einlagen ist im selben Zeitraum ebenfalls eine signifikante Besserung zu erkennen. Zwischen der ersten und zweiten Nachuntersuchung gab es keinen signifikanten Unterschied. In der Placebogruppe zeigte sich zwischen der Ausgangsmessung und der ersten Nachuntersuchung eine leichte und von Ausgangsmessung zur zweiten Kontrolluntersuchung eine deutliche jeweils signifikante Verbesserung der Funktion.

### EQ-5D

Im Teil b der Abb. 3 werden Ergebnisse des EQ-5D dargestellt. In der Gruppe der mit sensomotorischen Schuheinlagen versorgten Patienten zeigte sich eine leichte, aber signifikante Verbesserung des subjektiven Wohlbefindens zwischen der Ausgangsmessung und der ersten Nachuntersuchung, zwischen Ausgangsmessung und zweiter Nachuntersuchung, sowie zwischen erster und zweiter Nachuntersuchung.

In der Gruppe mit stützenden Schuheinlagen ergab sich eine signifikante Verbesserung des subjektiven Wohlbefindens zwischen der Ausgangsmessung und beiden Nachuntersuchungen. Zwischen erster und zweiter Nachuntersuchung zeigte sich keine signifikante Veränderung des subjektiven Wohlbefindens. Bei Verwendung von Placeboeinlagen konnte zwischen der Ausgangsmessung und der ersten Nachuntersuchung keine Verbesserung erreicht werden, aber zwischen der Ausgangsmessung und der zweiten Nachuntersuchung zeigte sich eine signifikante Verbesserung.

### NRS

**Abb. 4:**
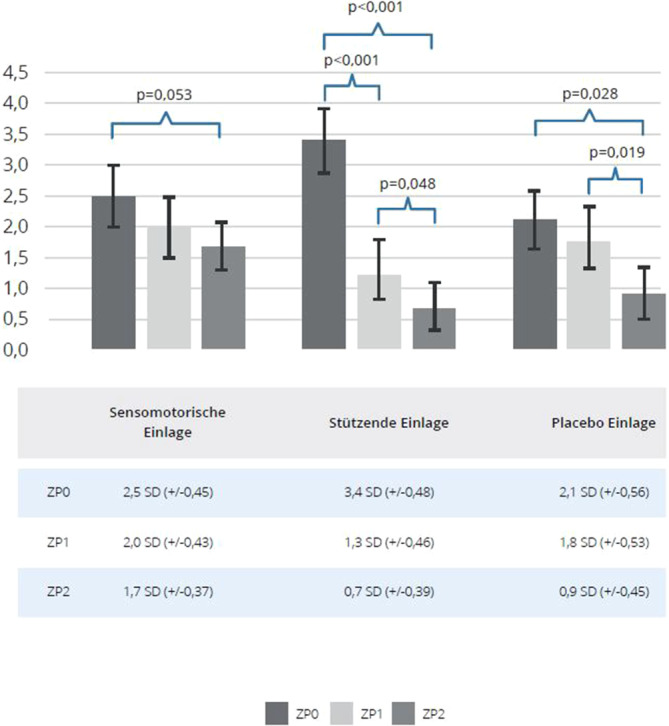
Vergleich der Numerischen Rating-Skala (NRS; dimensionslos) bei allen Studienarmen im Verlauf; *ZP0 *Ausgangsmessung, *ZP1 *1. Follow-up, *ZP2 *2. Follow-up, Mittelwert mit Standardabweichung (*SD*)

Die Ergebnisse hinsichtlich der Schmerzwahrnehmung zeigt Abb. 4. Die Anwendung sensomotorischer Einlagen führte lediglich zu einer tendenziellen Verringerung des Schmerzniveaus zwischen der Ausgangsmessung und der zweiten Nachuntersuchung. Bei Nutzung stützender Schuheinlagen konnte eine hochsignifikante Verringerung der Schmerzsymptomatik zwischen der Ausgangsmessung und der ersten sowie zweiten Nachuntersuchung gezeigt werden. Zudem zeigte sich eine signifikante Verbesserung zwischen der ersten und der zweiten Nachuntersuchung. In der Kontrollgruppe wurde keine signifikante Verbesserung zwischen der Ausgangsmessung und der ersten Nachuntersuchung gesehen. Es zeigte sich aber eine signifikante Verbesserung zwischen Ausgangsmessung und zweiter Nachuntersuchung. Signifikante Verbesserungen der Symptome wurden zudem zwischen der ersten und der zweiten Nachuntersuchung beobachtet.

### Auflagefläche (statisch)

**Abb. 5:**
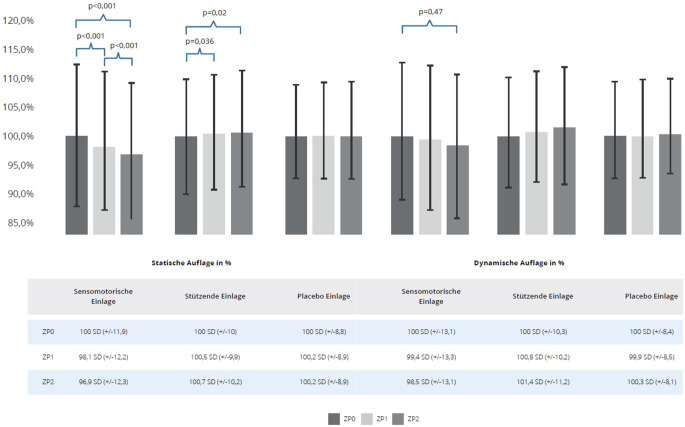
Statische Auflagefläche (*links*) und dynamische Auflagefläche (*rechts*) in Prozent für alle Studienarme (sensomotorische Einlage, stützende Einlage und Placeboeinlage); *ZP0 *Ausgangsmessung, *ZP1 *1. Follow-up, *ZP2 *2. Follow-up, Mittelwert mit Standardabweichung (*SD*)

Bei Nutzung sensomotorischer Schuheinlagen zeigte sich eine signifikante Verkleinerung der Auflagefläche im Bezug zur Ausgangsmessung bei allen Nachbeobachtungen (Abb. 5 [links]). Stützende Einlagen führten zu einer signifikanten Vergrößerung der Auflagefläche zwischen der Ausgangsmessung und beiden Nachuntersuchungen. Placeboeinlagen führten zu keiner Zeit zu einer signifikanten Veränderung der Auflagefläche.

### Auflagefläche (dynamisch)

Bei Verwendung sensomotorischer Einlagen verkleinerte sich in der mittleren Standphase die Auflagefläche zwischen der Ausgangsmessung und der zweiten Nachuntersuchung signifikant (Abb. 5 [rechts]). In der Gruppe der stützenden Einlagen vergrößerte sich die Auflagefläche zwischen der Ausgangsmessung und der zweiten Nachuntersuchung nicht signifikant. In der Placebogruppe zeigte sich zu keiner Zeit ein signifikanter Unterschied der Auflagefläche.

### VI

**Abb. 6:**
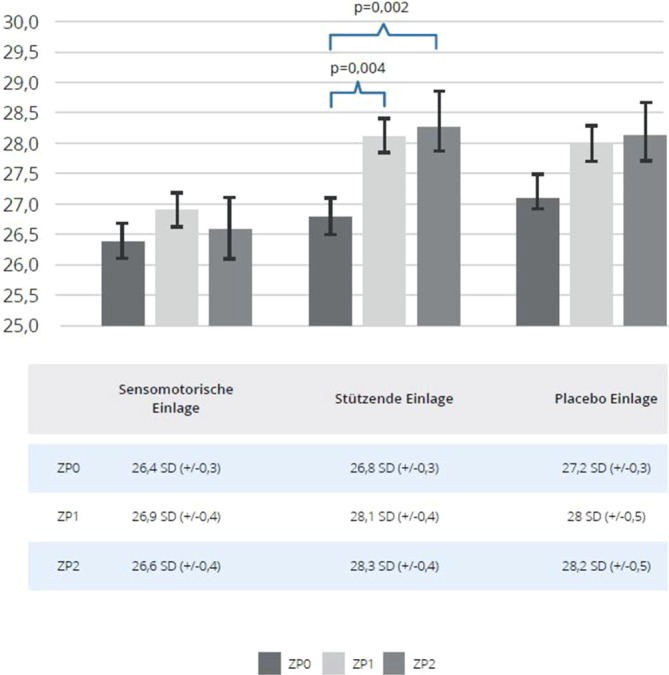
Valgus-Index (VI) bei allen Studienarmen (sensomotorische Einlage, stützende Einlage und Placeboeinlage); *ZP0 *Ausgangsmessung, *ZP1 *1. Follow-up, *ZP2 *2. Follow-up, Mittelwert mit Standardabweichung (*SD*)

Eine signifikante Verbesserung des VI konnte in keiner Gruppe und zu keinem Zeitpunkt beobachtet werden (Abb. 6). In der Gruppe der stützenden Schuheinlagen kam es zu einer signifikanten Verschlechterung des VI im Verlauf (Abb. 6).

**Abb. 7:**
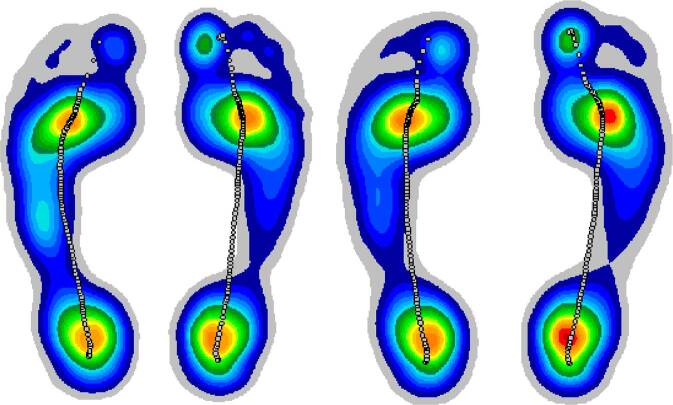
Dynamische pedobarographische Messung mit Drucklinie während der mittleren Standphase (maximale Auflagefläche); *links* Ausgangsmessung, *rechts* 2. Follow-up

Die Abb. 7 zeigt beispielhaft die Reduktion der Auflagefläche bei Anwendung einer sensomotorischen Einlage zusätzlich zum Fußübungsprogramm während der mittleren Standphase im Vergleich von Ausgangsmessung zur 2. Nachuntersuchung.

### Compliance

Die durchschnittliche Tragedauer der Einlagen in allen Gruppen und über die gesamte Zeit lag bei 7,9 (±2,2) Stunden am Tag. Das Training wurde nach Häufigkeit pro Woche abgefragt. Es erfolgte durchschnittlich 3,8-mal (±3,4) pro Woche. Es traten im Vergleich zwischen den Gruppen keine signifikanten Unterschiede bei Trainingshäufigkeit und Tragedauer auf.

## Diskussion

Es wird angenommen, dass ein Knicksenkfuß durch Überlastung der Unterschenkel- und Fußmuskulatur sowie des Bandapparates und daraus resultierender Fehlbelastung sowie Fehlstellung zu lokalen Beschwerden, Knieschmerzen, Fersensporn, Bandscheiben- und Rückenproblemen führen kann [2, 5, 7, 20, 21]. Die Behandlung mit stützenden Einlagen stellt bisher den Standard der konservativen Therapie dar [2, 3, 9, 22]. Hierbei zeigten sich positive Effekte in Bezug auf Schmerz und Funktion [23, 24]. Durch sensomotorische Einlagen konnten zwar Effekte auf die Unterschenkelmuskulatur bei Erwachsenen nachgewiesen werden, eine klinische Auswirkung auf Parameter wie Schmerz oder Funktionalität ist bisher jedoch noch nicht belegt [21]. Sensomotorische Einlagen haben Auswirkungen auf die Muskelaktivität des Unterschenkels [25].

Es konnte sowohl durch die stützenden als auch durch die sensomotorischen Einlagen in den subjektiven Funktionsscores (FADI und EQ5D) eine Besserung der Beschwerden erreicht werden. Dies konnte in der Gruppe der stützenden Einlagen dabei schneller nachvollzogen werden, da hier vermutlich durch die passive Aufrichtung der Fußwölbung der auslösende Reiz direkt adressiert wurde. Durch die passive Abstützung konnte eine Verbesserung in Schmerz und Funktion erreicht werden, was auch andere Autoren berichteten [16]. Da sich eine Symptombesserung, wenn auch verzögert, ebenfalls in der Placebogruppe zeigte, ist anzunehmen, dass die parallel durchgeführten Übungen für die Fußmuskulatur ebenfalls zu einer Beschwerdelinderung beitrugen [26]. Ein Placeboeffekt wäre hier allerdings auch denkbar. Der Schmerz wurde anhand der Numerischen Rating-Skala ermittelt, wobei in der Gruppe der stützenden Einlage der größte Rückgang der Schmerzen mit hochsignifikanten Werten belegt wurde. In der Gruppe der sensomotorischen Einlagen wurde das Signifikanzniveau knapp verfehlt, aber es konnte eine relevante Schmerzreduktion dargestellt werden.

Die Ursache für den anfänglich schwächeren Effekt in der sensomotorisch versorgten Gruppe könnte man durch die fortwährend spürbare Stimulierung im Bereich der Fußsohle durch sensomotorische Einlagen erklären, da diese insbesondere initial unangenehme Empfindungen bis hin zu Schmerzen auslösen können [13]. Dies sollte bei weiteren Untersuchungen gezielt berücksichtigt und hinterfragt werden. Eine Verbesserung der Funktionalität und des subjektiven Wohlbefindens konnte aber ebenfalls erreicht werden und somit hat sich die Therapie mit sensomotorischen Einlagen auch bei Beschwerden durch Knicksenkfuß in der untersuchten Kohorte therapeutisch bewährt. Durch den positiven Effekt der Aufrichtung der Fußwölbung ergibt sich vermutlich ein biomechanisch präventiv wirkender Effekt, der in weiteren Untersuchungen näher beschrieben werden sollte.

Durch die Verkleinerung der Auflagefläche konnte funktionell eine Aufrichtung der Fußlängswölbung demonstriert werden. Ursächlich für eine Anhebung der Fußlängswölbung ist, gemäß des postulierten Wirkprinzips sensomotorischer Einlagen, eine gezielte Aktivierung der Muskulatur [11, 12].

Bei stützenden Einlagen konnte eine Verringerung der Muskelaktivität z. B. im Bereich des Musculus peroneus longus nachgewiesen werden [25, 27]. Als Folge wäre eine weitere Abflachung der Fußwölbung denkbar, was bei dieser Versorgung durch die gezeigten Daten (Zunahme der Auflagefläche und Verschlechterung des Valgus-Index) auch signifikant sichtbar wurde [23, 25, 27, 28]. Durch stützende Einlagen kommt es zwar zur passiven Aufrichtung und somit zu einer passiven Korrektur der Fehlstellung, jedoch auch durch Verminderung der Beanspruchung der gewölbestützenden Muskulatur zu einer Verschlechterung der Funktion, was auch die klinischen Symptome ohne Einlage verschlechtern könnte und einen bereits symptomatischen Knicksenkfuß verschlimmern könnte, was das Gehen ohne Einlagen erschwert [2, 10, 20, 29].

Fußgymnastik wird bei Beschwerden im Bereich der unteren Extremität und teilweise auch bei Rückenschmerzen erfolgreich eingesetzt [26]. Hier könnten durch die Kombination mit einer sensomotorischen Einlage möglicherweise ebenfalls positive Effekte erreicht werden. Bei der Gruppe der stützenden Einlagen konnte trotz der Fußgymnastik eine Vergrößerung der Auflagefläche beobachtet werden. Der positive Effekt der Fußgymnastik, der durch die Symptombesserung der Placebogruppe offenkundig wurde, kann mit stützenden Einlagen offenbar eliminiert werden, sodass eine Versorgung mit stützenden Einlagen zwar wirksam in der Verbesserung der Beschwerden ist, langfristig aber die Stabilität der Fußwölbung eher negativ beeinflusst und zu einer „Abhängigkeit“ von Einlagen führen kann, wohingegen nach effektivem Anschub der Muskelaktivität und Fortsetzung der Fußgymnastik, die sensomotorische Einlage eventuell nur temporär getragen werden müsste.

Eine Stärke der Methodik dieser Arbeit ist, dass durch den Einsatz von Uniformdienstschuhen eine gute Standardisierung möglicher durch das Schuhwerk verursachter Einflüsse erreicht werden konnte. Aufgrund dessen, dass die Einlagen im Dienstschuh angepasst wurden, wurde eine Nutzung der Einlagen in einheitlichen Schuhen sichergestellt. Aufgrund der Individualität der Versorgung mit Schuheinlagen und unterschiedlichstem Schuhwerk wäre eine solche Studie sonst nur sehr kostenintensiv durchführbar und hätte bei vergleichbarer Tragedauer auch eine ähnliche Aussagekraft [30]. Weitere Stärken sind der Versuch der doppelten Verblindung und die Möglichkeit auf immer wieder den gleichen Orthopädieschuhmachermeister zurückgreifen zu können.

Eine Limitation der Arbeit besteht in der fehlenden Nachverfolgung von 15 Patienten, die zu Nachkontrollen nicht erschienen und teilweise nicht erreichbar waren. Die Ursachen für das Fernbleiben waren so nicht in jedem Fall zu eruieren, könnten aber darin begründet sein, dass das Einzugsgebiet für die Patienten von Rostock bis Berlin und von Hagenow bis Torgelow reichte und teilweise erhebliche Anfahrtswege in Kauf genommen werden mussten. Weiterhin könnten negative Effekte (keine Verbesserung der klinischen Symptome und daraus resultierende Demotivation bezüglich der Therapieadhärenz) genauso wie positive Effekte (Therapieerfolg führt zu fehlender Motivation die zeitliche Belastung für die aufwendigen Nachuntersuchung wahrzunehmen) hierfür ursächlich sein. Weiterhin wurde versucht eine Verblindung zu erreichen und es wurde weder dem Patienten noch dem Untersucher mitgeteilt, welcher Behandlungsgruppe der Patient zugeordnet wurde. Trotz Versuch der größtmöglichen optischen Angleichung, kann nicht ausgeschlossen werden, dass Patienten die Art der Intervention erkennen konnten, was schwer vermeidbar war, sodass zwar der Versuch der Verblindung erfolgte, aber letztlich nicht zu 100 % gewährleistet werden kann, dass jeder Patient im Unklaren über die Therapiegruppe war, obwohl keine Information diesbezüglich erteilt wurde. Da der Untersucher keinen unmittelbaren Kontakt mit der Einlage hatte, war die Verblindung untersucherseits nicht eingeschränkt.

Die Durchführung des Fußmuskelübungsprogramm wurde mittels Abfrage quantifiziert. Hierbei zeigte sich eine breite Spannweite hinsichtlich der Compliance in allen Gruppen. Dies beeinträchtigt die Wertung des Effekts der Fußmuskelübungen. Da dies aber in allen Gruppen gleichermaßen der Fall war, können die Aussagen hinsichtlich des Einflusses der Einlagen weiter getroffen werden.

Auch wenn in dieser Arbeit erstmals indirekt positive Effekte auf die Fußstatik nachgewiesen wurden, bleibt Raum für die weitere Bearbeitung gezielter Fragestellungen. Aufgrund der Fallzahl konnten Einflüsse anderer Parameter, wie Geschlecht, Alter, Gewicht, sportliche Aktivität nicht weiter differenziert betrachtet werden.

Serafin et al. konnten einen deutlichen Unterschied beim Muskelaufbau und in der Bindegewebestruktur zwischen Frauen und Männern nachweisen, weswegen in einer zukünftigen Betrachtung die Effekte auch fokussiert geschlechtsspezifisch ausgewertet werden könnten [31]. In unserer Untersuchungsstelle (Facharztzentrum der Bundeswehr) ist der Anteil weiblicher Patienten strukturbedingt geringer.

## Fazit für die Praxis

- Stützende Einlagen führten bei Erwachsenen schneller zur Reduktion von subjektiven Beschwerden, vergrößerten aber signifikant die Auflagefläche und erhöhten signifikant den VI, erklärbar durch eine zunehmende Inaktivierung der gewölbestützenden Muskulatur
- Sensomotorische Einlagen erzeugten in Kombination mit Fußgymnastik eine bessere aktive, statische und dynamische Stabilisierung der Fußwölbung (Reduktion der Auflagefläche), wenngleich die Schmerzabnahme verzögert auftrat
- Fußgymnastik ohne wirksame Einlage führte zu keiner signifikanten Änderungen der Fußbiomechanik, aber auch zu einer Beschwerdereduktion und war damit effektiv, auch wenn der Wirkeintritt später erfolgte
- Aufgrund der Wirksamkeit von Fußübungen könnte eine temporäre Versorgung mit sensomotorischen Einlagen zum schnelleren Erreichen eines therapeutischen Effektes und späterer alleiniger Übungsdurchführung suffizient sein, wohingegen bei stützenden Einlagen eine Dauerversorgung notwendig erscheint

## Data Availability

DRKS, DRKS00028005. Registred 17.02.2022—retrospectively registered, https://drks.de/search/de/trial/DRKS00028005. Die erhobenen Datensätze können auf begründete Anfrage in anonymisierter Form beim korrespondierenden Autor angefordert werden. Die Daten befinden sich auf einem Datenspeicher der orthopädischen Klinik und Poliklinik des Universitätsklinikums Rostock.

## Abkürzungen

ANOVA: Analysis of variance
BMI: Body-Mass-Index
EQ-5D: EuroQol 5D
FADI: Foot and Ankle Disability Index
NRS: Numerische Rating-Skala
SD: Standardabweichung
VI: Valgusindex
ZP0: Ausgangsmessung
ZP1: 1. Follow-up
ZP2: 2. Follow-up
